# Does the green finance policy affect the efficiency of corporate investment? Evidence from China’s green finance reform and innovation pilot zones

**DOI:** 10.1371/journal.pone.0313861

**Published:** 2024-11-15

**Authors:** Hongxin Wang, Rong Shao

**Affiliations:** Department of Business Administration, Liaoning Technical University, Huludao, Liaoning, China; Southwestern University of Finance and Economics, CHINA

## Abstract

In the context of promoting the orderly expansion of capital investment and rational allocation of resources to achieve green and circular economic development. Green finance, as a new engine to promote the sustainability of enterprises, holds significant importance in exploring the positive effect of green finance policies on optimizing the investment decisions of enterprises and guiding them to efficiently utilize their resources to maximize value creation. Using A-share listed companies in Shanghai and Shenzhen from 2012 to 2022 as the research sample, we apply the Difference-in-Differences (DID) method to test the impact of the green finance reform and innovation pilot zones (2017) on the investment efficiency of enterprises in the pilot regions. We also adopt a two-step method to test the mechanisms of financial resource misallocation and agency costs. The study reveals that the green finance policy significantly enhances the investment efficiency of enterprises in the pilot areas. Financial resource misallocation and agency costs are important influence mechanisms. Drawing on resource allocation theory and agency theory, the study concludes that the green finance policy alleviates financial resource misallocation by directing financial resources toward high-efficiency enterprises. Moreover, the policy effectively reduces agency conflicts caused by power separation and information asymmetry, ensuring that enterprises can maximize the benefits of their investments. Heterogeneity analysis shows that non-state-owned enterprises and low-tech innovative enterprises in the pilot areas have disadvantages in terms of capital stock and loan credit, so the policy can improve their investment efficiency effectively. Based on these findings, we recommend that to leverage the positive effects of the green finance policy, it is essential to enhance the guiding role of the government, strengthen market mechanisms, and bolster corporate initiatives. This study complements the research on the economic effects of location-orientated comprehensive green finance policies on enterprises, considering the dual aspects of resource allocation efficiency and corporate governance, and makes up for the shortcomings of the existing literature. The study’s conclusions offer valuable insights for enhancing green finance to support enterprises in achieving efficient production.

## 1 Introduction

The concept of harmonious coexistence of humans and nature transforms the relationship between ecological protection and economic growth from a contradictory relationship to a dialectical unit, the coordination between protection and development has pushed green and low-carbon development into the forefront of contemporary issues and made it a crucial component for the sustainable growth of China’s economy [1]. Green and low-carbon development has established ecological protection as a red line for China’s economy, with a commitment to breaking the constraints of resource misallocation. By promoting improvements in resource allocation efficiency and the orderly expansion of capital investment, it gradually shifts away from the crude economic development mode of blindly expanding scale and making inefficient investments, moving instead to a green and circular economic development mode to realize the goal of high-quality development [2]. The 14th Five-Year Plan points out that the institutional mechanism of financial support for the real economy should be constructed vigorously. In the development of enterprises’ green and low-carbon, end-of-pipe treatment plays an important role, but it has certain limitations, therefore, it is also crucial to cooperate with financial instruments to address the issue from the source. Exploring how to guide financial support through policy to support enterprises to efficiently use resources for green transformation and further drive the high-quality development of the real economy is highly significant.

Green finance, as a valuable complement to traditional finance and environmental regulation, considers both environmental and economic benefits and coordinates the contradiction between financial resource allocation and the negative externality of pollution [3, 4]. The Equator Principles emphasize that financial institutions should focus on the environmental impact of investment projects when issuing credit, green finance directs economic agents to pay attention to ecological and environmental protection, utilize resources efficiently, and achieve sustainable development through differentiated financial resource allocation. Enterprises transform high-return investment opportunities into actual investment through rational allocation of resources, and this investment behavior not only significantly contributes to the value creation and sustainable development of enterprises [5], but also contributes to the transformation and upgrading of China’s economy [6]. According to MM theory, under perfect market conditions, the enterprise’s investment motivations and decisions are completely determined by investment opportunities, independent of capital structure [7]. However, a perfect market does not exist, and inefficient investment is common among enterprises, resulting in issues such as default risk and stock price collapse [8]. Consequently, improving corporate investment efficiency has always been a crucial issue for enterprises to realize sustainable development. The development of green finance offers new opportunities for corporate investment decision-making and value creation.

The literature relevant to this research can be categorized into three primary streams. The first stream examines the economic and environmental effects of green finance from both macro and micro perspectives. From a macro perspective. The economic effects emphasize the influence of green finance on economic growth, including industrial structural upgrading, urban economic resilience, and high-quality economic development [9–11]. The environmental effects focus on the sustainability of the environment, including carbon emissions, energy utilization efficiency, and environmental pollution governance. Jahanger et al. [12], taking ASEAN countries as their research sample, assert that green finance is crucial for reducing carbon emissions and attaining environmental sustainability. Agrawal et al. [13] and Li et al. [14] further argue that integrating green finance with green technological innovations can be more effective in achieving sustainable development and combating climate change. From a micro perspective. Research on the economic effects mainly focuses on corporate debt financing and high-quality development. Some scholars argue that green finance has a financing penalty effect on enterprises with negative environmental information and a financing incentive effect on green enterprises [2, 15]. The environmental effects are mainly discussed in the promotion of green innovation, green transformation, and ESG performance [4, 16, 17]. The underlying mechanisms are mainly explored in the resource allocation mechanism and information-driven mechanism. Scholars suggest that green finance increases the cost of debt financing for heavy polluters through differentiated resource allocation [18, 19], while reducing financing pressures on green enterprises [20]. Additionally, green finance improves information disclosure mechanisms, allowing resources to be tilted in favor of green enterprises through information-driven channels [21, 22]. The second stream of literature explores the factors influencing the investment efficiency of enterprises, with existing studies primarily concentrating on both the internal and external environments influencing these efficiencies. The internal environment mainly focuses on two factors, principal-agent problem and information asymmetry, including agency conflicts between shareholders and management, conflicts between large and small shareholders, and the information asymmetry that arises from agency conflicts [23]. The external environment mainly focuses on the adverse selection issues caused by information asymmetry and external policy systems. For example, scholars argue that the greater the distortion in resource allocation, the further enterprises deviate from the optimal investment decision [24, 25]. Additionally, some scholars suggest that government intervention plays a vital role in the investment efficiency of the enterprise [26], particularly through the enactment of policies such as environmental policy, monetary policy, industrial policy, etc. [27–29]. In recent years, due to the effect of COVID-19 and the uncertainty of the economic environment, more and more investors have recognized the expected value of sustainable assets, and investment in sustainable projects has risen, drawing the attention of many scholars [30, 31]. The third stream of literature explores the impacts between green finance and corporate investment activities. There are three main views. The first argues that green finance can enhance corporate investment efficiency [32]. The second argues that green finance reduces corporate investment efficiency [33, 34]. The third viewpoint indicates a nonlinear relationship between the two [35].

In summary, numerous scholars focus on the microeconomic impacts of various green finance policies, particularly through the perspective of corporate financing. Among the limited literature that takes the perspective of investment efficiency, several issues remain: there are divergent conclusions, insufficient exploration of mechanisms, and the study object focuses on heavy polluters. The ultimate purpose of green finance is not to impact corporate financing, nor is it to force inefficient enterprises out of the market, but to guide enterprises to efficiently utilize resources for investment through financial means, and to shift from a scale-driven investment model to an efficiency-driven investment model, accelerate enterprise transformation and upgrading, so it is of great significance to pay attention to the impact of green finance on corporate investment efficiency. Moreover, in the literature exploring corporate investment efficiency from an external policy perspective, fewer studies investigate the effects of green finance policies, and most of them reflect the effect of the implementation of comprehensive green finance from a singular perspective of green credit, and the choice of policies is more one-sided.

This study Uses the 2017 green finance reform and innovation pilot zones (hereafter referred to as the green finance policy) as a quasi-natural experiment, selecting A-share listed companies in Shanghai and Shenzhen from 2012 to 2022 as samples, and applying a DID model to explore the effects of the green finance policy on corporate investment efficiency in pilot regions. This study also analyzes the mechanisms of financial resource misallocation and agency costs. Based on the analysis of heterogeneity, investment efficiency varies among different enterprises due to their differing sensitivities to the policy.

The contributions are as follows:

Firstly, from the standpoint of corporate investment efficiency, it complements the literature regarding the effects of location-oriented comprehensive green finance policies on economic outcomes. The study considers both green enterprises and heavy polluters, which are most profoundly influenced by the policy, and the heterogeneous effects on these two types of enterprises are separately explored, concluding that the green finance policy improves the investment efficiency of enterprises in the pilot areas. The study offers theoretical support for green finance’s role in facilitating efficient resource utilization and the green transformation of enterprises. The study also reveals that the green finance policy has different policy impacts based on different types of companies, providing reference and inspiration for green finance to maximize its effectiveness according to local conditions.

Secondly, Theoretical insights are offered regarding the transmission mechanisms by which green finance policy influences corporate investment efficiency. Drawing on resource allocation theory and agency theory, and considering the dual pathways of resource allocation efficiency and corporate governance, the transmission role of financial resource misallocation and agency costs in the connection between the green finance policy and corporate investment efficiency is clarified through empirical tests. The study analyses the two main causes of financial resource misallocation: government intervention and rationing discrimination, and the two main drivers of agency costs: the separation of powers and information asymmetry. the positive effects of the green finance policy on alleviating financial resource misallocation and reducing agency costs are analyzed. Based on these two mechanisms, the study bridges the gap in the literature concerning corporate investment efficiency and provides dual empirical evidence for the positive impact of the green finance policy.

The remainder of this paper is organized as follows: the second section describes the policy background, theoretical foundations, and research hypotheses; the third section provides the research design and data sources; the fourth section encompasses correlation analysis, descriptive statistics, benchmark regression, and robustness tests; the fifth section further analyzes the impact mechanisms and enterprise heterogeneity; the sixth part is a discussion; and the seventh section summarizes the study’s findings.

## 2 Policy background and research hypothesis

### 2.1 Policy background

Before 2015, China’s green finance development primarily focused on the promotion and deployment of green credit, which was market-driven and aimed to increase the cost for enterprises with high emissions and significant pollution. In 1995, the Central Bank of China first pointed out that financial institutions should consider environmental protection in their lending practices. In 2012, the “Green Credit Guideline” was issued, outlining the strategic direction for green credit development. in 2014, CBRC formulated and implemented “Key Evaluation Indicators for the Implementation of Green Credit” to safeguard the development of green credit. After 2015, China entered a stage of large-scale green finance development, gradually establishing a top-level design. In 2016, the “Guiding Opinions on Building a Green Financial System” was issued, offering the initial formal definition of green finance and outlining a comprehensive framework for developing a green financial system. In 2017, the construction of green finance reform and innovation pilot zones was carried out, with a five-year trial period in five provinces to develop green finance from the ground up. The green finance policy aimed to motivate financial institutions to broaden their green finance initiatives and assist enterprises in issuing green financial instruments. Each of the five provinces had different economic, ecological, and institutional environments, leading to different developmental objectives and the exploration of replicable experiences. Zhejiang Province focused on using green finance to support the upgrading of traditional industries, Guangdong Province emphasized creating a green finance market aligned with its economic development goals, Guizhou and Jiangxi Provinces focused on using green finance to support ecological development, and the Xinjiang Uygur Autonomous Region focused on using green finance to support the development of agriculture and clean energy, to expand westward. The implementation of the green finance policy has greatly contributed to the large-scale development of green finance and improving the green finance standard system. In recent years, relevant rules and guidelines for the development of green finance have been issued one after another, such as “Green Industry Guidance Catalogue (2019)” and “China Green Bond Principles (2022)”, which continue to improve the top-level design and accumulate practical experience. So far, the development of green finance in China has been at the forefront globally, and its influence has been increasing. By 2022, the most mature segment of green finance, green credit, has surpassed 22 trillion yuan in outstanding balances, ranking first. Green bonds, the other mainstay of green finance, have continued to gain momentum and are in a second-place position.

### 2.2 Theoretical analysis and research hypothesis

#### 2.2.1 Green finance and corporate investment efficiency

The theory of externalities states that when one economic agent has a non-market price impact on another economic agent, positive or negative externalities arise, requiring intervention through taxes or subsidies to internalize these effects [36, 37]. Negative environmental externalities distort resource allocation, leading to inefficient production and investment for firms. The green finance policy has significant positive externalities, and internalizes negative environmental externalities through policy guidance and incentive constraint effects, thereby promoting more efficient production and investment by enterprises. Based on external pressures. First, the green finance policy strengthens the market’s leading role. From the viewpoint of the supply and demand mechanism, financial institutions prudently assess the environmental performance of enterprises and potential environmental risks by establishing environmental credit evaluation criteria. This enables resource allocation to be aligned with market supply and demand, forcing enterprises to pay attention to sustainable development and make efficient investments. From the perspective of the information transfer mechanism, financial institutions use the environmental information of enterprises as an important risk assessment tool, reducing the potential risks of enterprises concealing environmental pollution and curbing the “greenwashing” behavior of enterprises [38]. This prompts enterprises to channel funds into sustainable projects, thus improving investment efficiency. Second, the green finance policy strengthens the government’s regulatory function. Negative environmental externalities may lead to market failure [39], and the government increases the supervision, rewards, and punishments for both financial institutions and enterprises. Incorporating green credit performance into the macro-prudential assessment, the government incentivizes financial institutions to prioritize green factors in risk management and asset allocation. At the same time, it increases the political cost for enterprises that prioritize profit over environmental responsibility by internalizing negative environmental externalities, thereby prompting enterprises to improve investment efficiency. Based on internal motivation. First of all, Strict environmental requirements and financial regulations make enterprises face high environmental violation penalties, when the cost of non-compliance outweighs the cost of compliance, enterprises will carefully consider investment decisions and increase investment in green projects, thus reducing fines due to violations. Secondly, a good green reputation, as a substitute for conveying corporate environmental information [40], helps to send good development signals. The green finance policy curbs inefficient investments by mitigating reputation risk [41]. To cope with the policy’s impact, enterprises will efficiently utilize their resources to boost the development of green industries, improve corporate investment efficiency, and increase the trust of financial institutions and the government by building a green reputation.

Hypothesis 1 (H1): The green finance policy can significantly improve the investment efficiency of enterprises in pilot zones.

#### 2.2.2 Green finance, financial resource misallocation and corporate investment efficiency

Resource allocation theory states that resource allocation reaches Pareto optimality only when financial resources flow to efficient subjects [42, 43]. Resource allocation inefficiency often manifests as an imbalance between supply and demand. Rational allocation of financial resources is crucial for maximizing investment efficiency, as it is a key component of resource allocation and fundamental to corporate investment activities. The main reasons for financial resource misallocation are government intervention and rationing discrimination [44].

From the perspective of government intervention. The theory of financial repression holds that in developing countries, governments promote economic development by intervening in financial markets. The allocation of financial resources is often affected by government preferences [45], leading to inefficient enterprises receiving funds while efficient enterprises may struggle to secure investment. In the context of green development, to solve the misallocation problem of financial resources flowing to enterprises with better hard conditions but poorer efficiency, the green finance policy is committed to promoting the organic combination of government intervention and market mechanisms. Through cross-sectoral collaboration, the pilot zones have set up leadership groups to enhance the strategic framework of green finance and established sound supporting measures, including financial support, information platform construction, and administrative supervision. These efforts aim to ensure financial institutions allocate resources rationally and urge enterprises to utilize resources to create value efficiently.

From the perspective of rationing discrimination. Equilibrium credit rationing theory states that in markets with incomplete information, when the demand for resources exceeds supply, financial institutions will adopt non-interest-rate criteria to select borrowers, which leads to the difficulty of enterprises with poor conditions but better efficiency in obtaining financial resources to safeguard their daily investment activities [46]. The distortion of resource rationing leads to under-investment and over-investment within enterprises [47]. Compared with the mature green finance in Western developed countries, the autonomous environmental awareness of enterprises in developing countries, led by China, has not yet been fully established, which makes it difficult to take the initiative to internalize environmental responsibility [48], so the focus of China’s green finance development is mainly on the differentiated allocation of financial resources under the same price, and tilting the financial resources to the high-efficiency and low-pollution enterprises [21].

Green enterprises, heavily impacted by the green finance policy, face significant maturity misallocation problems. Their investments mainly involve projects with long payback periods, unknown returns, and higher risks, such as renewable energy and low-carbon technologies [32]. While financial institutions typically prefer short-cycle and high-return projects when considering the allocation of financial resources, the misallocation of financial resources mainly causes green enterprises to face the dilemma of insufficient funds in their investment activities, which hampers both the scale and efficiency of their investments. The green finance policy guides financial resources towards green enterprises, alleviating financial resource misallocation and encouraging investment in low-emission, high-efficiency green industries. From an economic policy perspective, the green finance policy expands financing channels to encourage proactive investments by green enterprises. Financial institutions are actively promoting the innovation of green financial products, while social capital is encouraged to set up green industry funds. This provides green enterprises with external funds to meet their investments by reallocating financial resources and reducing the possibility of over-investment by enterprises in a single project. At the administrative level, the policy strengthens access audits and market supervision of financial institutions, such as using green bank ratings as an important factor in institutional assessments, ensuring financial resources are reasonably allocated to green enterprises, and creating a good ecological environment for efficient investment.

Heavily polluting enterprises, which are significantly affected by the green policy, are usually favored by financial institutions for resource allocation, primarily due to their scale, total assets, and repayment ability, rather than their environmental performance and investment benefits. This irrational resource allocation leads heavily polluting enterprises to rely excessively on low-cost funds, making them overly blind when making investment decisions, and leading to inefficient investments that ignore environmental benefits [49]. The green finance policy produces an investment penalty effect on heavily polluting enterprises by restricting the input of financial resources [50]. Financial institutions consider environmental risk an important factor in allocating financial resources, which limits the flow of financial resources to inefficient production capacities and reduces the supply of long-term debt that exacerbates over-investment. Additionally, the increased transparency of environmental information on heavily polluting enterprises has made it inevitable that enterprises will be condemned at the moral level of society and penalized by government departments. The transmission of unfavorable signals reduces the willingness of financial institutions to provide financing to heavily polluting enterprises. As a result, heavily polluting enterprises must scale back production inputs and expand environmental protection investments [51]. Under the condition of limited financial resources, on the one hand, heavy polluters will optimize their investment decisions and prioritize green projects with higher potential, avoiding over-investment and irrational expansion. On the other hand, to obtain the necessary financial support, they will improve their environmental performance, enhance the efficiency of resource utilization, and promote more efficient investment. It is noteworthy that under the differentiated financial resource allocation, heavy polluters in green transformation face heightened financing constraints., Commercial credit financing can serve as an alternative to alleviate under-investment caused by the punitive effect of financing in enterprises in transition [52].

Hypothesis 2 (H2): The green finance policy can alleviate financial resource misallocation and thus improve the investment efficiency of enterprises in pilot zones.

#### 2.2.3 Green finance, agency costs and corporate investment efficiency

According to agency theory, the agency conflict between corporate management and corporate shareholders due to the separation of ownership and operational rights and resulting information asymmetry is the fundamental factor affecting corporate investment efficiency [53, 54]. The green finance policy reduces agency costs and promotes efficient investment by enterprises in pilot areas.

The direct cause of agency conflict is the separation of two rights. Agency theory states that the separation leads to a deviation of goals between principals and agents, and due to limited monitoring, corporate management makes investment decisions that are detrimental to sustainable development and shareholder interests in pursuit of their interests. These unfavorable investment decisions can be categorized as follows: over-investment by management in pursuit of private interests, leading to indiscriminate scaling of investment; and under-investment by management in pursuit of private costs, thus avoiding the risk of green inputs, and securing the position of the manager by abandoning investment projects with positive NPV [55]. The green finance policy addresses both types of unfavorable investment decisions, with the government and financial institutions implementing different measures for over-investment and under-investment. For over-invested enterprises, the green finance policy prompts financial institutions to enhance risk assessment and monitoring by establishing robust green finance risk mechanisms and incorporating enterprises’ environmental conditions into the national credit-sharing information platform. This approach leverages the governance effects and the supervisory effects of financial institution to limit the disposable capital of such enterprises [56]. Management reduces the indiscriminate use of free cash flows to sustain the company’s survival and growth, thereby alleviating over-investment and resource allocation distortions, which promotes efficient investment [57]. For under-invested enterprises, localities have enacted relevant preferential policies on finance, taxation, and loans for green financial products. localities have also issued diversified financial products and innovative green financial tools to improve the investment return rates of under-invested enterprises, and collaborate in establishing mechanisms for risk compensation related to green project investments and financing, thereby mitigating investment risks. Consequently, it improves the investment profitability of under-invested enterprises [58], allowing the management of enterprises to avoid the risks associated with green investment while maintaining professional status and seizing investment opportunities to improve investment efficiency.

The fundamental cause of agency conflicts is information asymmetry. First of all, in situations of significant information asymmetry, the selective disclosure of information by enterprises makes it difficult to accurately assess the financial status and operating results of enterprises, which can easily lead to missed investment opportunities. The green policy has accelerated the development of a green financial information-sharing platform and improved the legal framework for environmental information disclosure. This platform consolidates and shares corporate information on green projects and environmental governance, enabling management to present the green development of the enterprise through environmental information disclosure. Such disclosure is conducive to reducing the problem of adverse selection and the information risk of enterprises, encouraging enterprises to seize the investment opportunities and invest efficiently [59]. Secondly, within the framework of agency theory, when there is no external pressure, even effective environmental regulation may render credit allocation between the principal and agent ineffective [4], which is not conducive to investment efficiency. Based on this, the green finance policy standardizes the review mechanism of financial institutions on corporate environmental information disclosure and raises the costs associated with inaccurate and illegal disclosure by corporations [38]. This mechanism leads corporate environmental information to become more transparent and makes the transmission of information more timely and more accurate. On the one hand, shareholders can more effectively evaluate the sustainability of enterprises and the feasibility of investment decisions, thereby improving the accuracy and efficiency of investment. On the other hand, it strengthens shareholders’ supervision of corporate management, enabling them to better understand and supervise management’s investment activities through environmental information. If shareholders are skeptical about the investment behavior of the managers, it can seriously damage the reputation of the management. In order to maintain the reputation of the management, the management may opt to give up the short-term interests and take the initiative to assume social responsibility, which ultimately reduces agency costs and allows management to identify investment opportunities and execute highly efficient investments.

Hypothesis 3 (H3): The green finance policy can reduce corporate agency costs and thus improve the investment efficiency of enterprises in pilot zones.

## 3 Research design

### 3 1 Data sources

Firstly, all the listed companies in Shanghai and Shenzhen A-shares from 2012 to 2022 were selected as the research samples. As the explained variable is a dummy variable, the degree of missing data was mainly determined by the explanatory and control variables. After measuring all variables, a total of 4,050 sample companies and 26,649 company-year observations were identified. Secondly, data filtering according to the criteria in Table 1, resulted in a final sample of 2,204 companies and 19,649 company-year observations. Among these, the treatment group consisted of 667 companies with 5,859 company-year observations, while the control group comprised 1,537 companies with 13,790 company-year observations. Corporate base information and financial data from the CSMAR database (Chinese Research Data Services), and the corporate patent data from the CNRDS database (Chinese Research Data Services). Using STATA17 software to analyze and process data.

**Table 1:** Sample screening.

Screening Steps	Screening Criteria	Screening Reasons	Exclusion of Company-Year Observations (nos.)
1	Exclusion of financial enterprises	Based on accounting peculiarities	911
2	Excluding ST, ST*, and PT enterprises	To ensure data stability	1466
3	Excluding enterprises with more missing values	Ensure data are available before and after the policy implementation year and guarantee empirical accuracy	4623
4	Perform 1% winsorization on continuous variables.	Avoidance of bias due to extreme values	0

### 3.2 Model construction

To test the relationship between the green finance policy and corporate investment efficiency, this paper adopts the DID method. As the most widely used method for evaluating policy effects, the DID method categorizes samples affected by policy shocks as the treatment group and those unaffected as the control group. The policy effect was determined by comparing the differences between the treatment and control groups before and after the policy implementation. The application of the DID method is based on two premises: first, the treatment group should be directly influenced by the policy whereas the control group remains unaffected; and second, the parallel trend test must be satisfied. The DID method can alleviate the endogeneity problem and reduce the selection bias, but it is difficult to completely eliminate the influence of other factors on the results, and there are high requirements on the total amount of data. The DID model is constructed as follows.

$$Absinv_{i,t}=\alpha_{0}+\alpha_{1}TEST_{p}\times GFP_{t}+\rho X_{i,t}+\mu_{p}+\delta_{j}+\theta_{t}+\varepsilon_{i,p,j,t}$$ (1)

Where i, p, j, and t represent company, province, industry, and year, respectively. Absinv represents inefficient investment, serving as a proxy variable for investment efficiency; the lower the value, the greater the corporate investment efficiency; TEST represents the group dummy variable; GFP represents the time dummy variable; TEST×GFP represents interaction term; X represents a set of control variables affecting investment efficiency; μ, δ, and θ represent province, time and industry fixed effects, respectively; ε represents a random perturbation term. Firm-level clustering is performed for t-values, and due to the incorporation of three types of fixed effects, separate terms for TEST and GFP are omitted to reduce multicollinearity.

### 3.3 Variables

#### 3.3.1 Explained variable

Inefficient investment (Absinv). We utilize the expected investment model to quantify inefficient investment. Specifically, the difference between the actual investment expenditure of the enterprise and the expected investment expenditure of the enterprise is taken as the absolute value and recorded as the inefficient investment. This measure is used as the proxy variable for investment efficiency. Referring to [60], the following model is developed.


$$\begin{array}{c} Invest_{\text{i},\text{t}}=\beta_{0}+\beta_{1}Growth_{i,t-1}+\beta_{2}Lev_{i,t-1}+\beta_{3}Cash_{i,t-1}+\beta_{4}Age_{i,t-1}+\beta_{5}Size_{i,t-1}\\ \;+\beta_{6}\text{Re}turn_{i,t-1}+\beta_{7}Invest_{i,t-1}+\delta_{i}+\theta_{t}+\nu_{i,t} \end{array}$$ (2)

The level of investment (Invest), which is measured by the ratio of capital expenditures, minus proceeds from the liquidation of assets and current depreciation, to opening assets. The rest of the variables represent: Growth represents investment opportunity, denoted by TobinQ; Lev represents gearing ratio; Cash represents cash flow position; Age represents the age of the firm; Size represents the size of the firm; Return represents stock return; ν represents residual, with a one-year lag of the dependent variable. In addition to investment expenditures due to normal business operations, there are abnormal business investment expenditures, which are represented by the residual. The level of inefficient investment (Absinv) is defined as the absolute value of the residual, When the absolute value of the residual is smaller, it indicates a lower level of inefficient investment (Absinv) and greater corporate investment efficiency. When the residual is greater than 0, it signifies that the enterprise is experiencing over-investment (Overinv). In this case, the smaller the residual, the lower the degree of over-investment. When the residual is less than 0, the enterprise is experiencing under-investment (Underinv). In this case, a smaller absolute value indicates a lower degree of under-investment.

#### 3.3.2 Explanatory variable

Green finance policy (TEST×GFP). Measured by the interaction term of two dummy variables. Taking the green finance policy issued in June 2017 as a quasi-natural experiment, 2017 is considered the first year of policy implementation. Before the policy’s issuance, GFP is coded as 0; post-issuance, GFP is coded as 1. The first batch of pilot provinces for green finance in 2017 were Zhejiang, Jiangxi, Guangdong, Guizhou, and Xinjiang Uygur Autonomous Region. Gansu Province was designated as the second batch of pilot provinces in 2019, and Chongqing continued as a pilot area in 2022. Taking into account the radiation and spillover effects of the promulgated documents and systems on neighboring areas, companies located in pilot provinces are designated as the treatment group with TEST coded as 1, while those in other provinces are designated as the control group with TEST coded as 0.

#### 3.3.3 Control variables

To account for other variables that may affect the explanatory variables, the following control variables were selected based on previous literature: the percentage of shares held by the top shareholder (Top), the percentage of independent directors (Indep), financial leverage (Lever), book-to-market ratio (BM), revenue growth rate (Grow), total asset turnover (ATO), age of the enterprise (AGE), earnings volatility (EQ). The variables are defined and measured in Table 2.

**Table 2:** Variable definition and measurement.

Variable Name	Variable Symbol	Variable Calculation Formula
Inefficient investment	Absinv	Calculated based on model (1)
Under-investment	Underinv	Calculated based on model (1)
Over-investment	Overinv	Calculated based on model (1)
Percentage of shares held by top shareholders	Top	Number of shares held by the largest shareholder/total number of shares
Percentage of independent directors	Indep	Number of independent directors/size of directors
financial leverage	Lever	Total liabilities/total assets
Book-to-market ratio	BM	Shareholders’ equity/company market capitalization
Revenue growth rate	Grow	Growth rate of operating income over the previous year
Total asset turnover	ATO	Operating income/total assets
Age of enterprise	AGE	Years of incorporation plus 1 to take the logarithm
Earnings volatility	EQ	Three-year volatility of EBITDA/total assets

## 4 Results

### 4.1 Correlation analysis and descriptive stats

**Fig 1:**
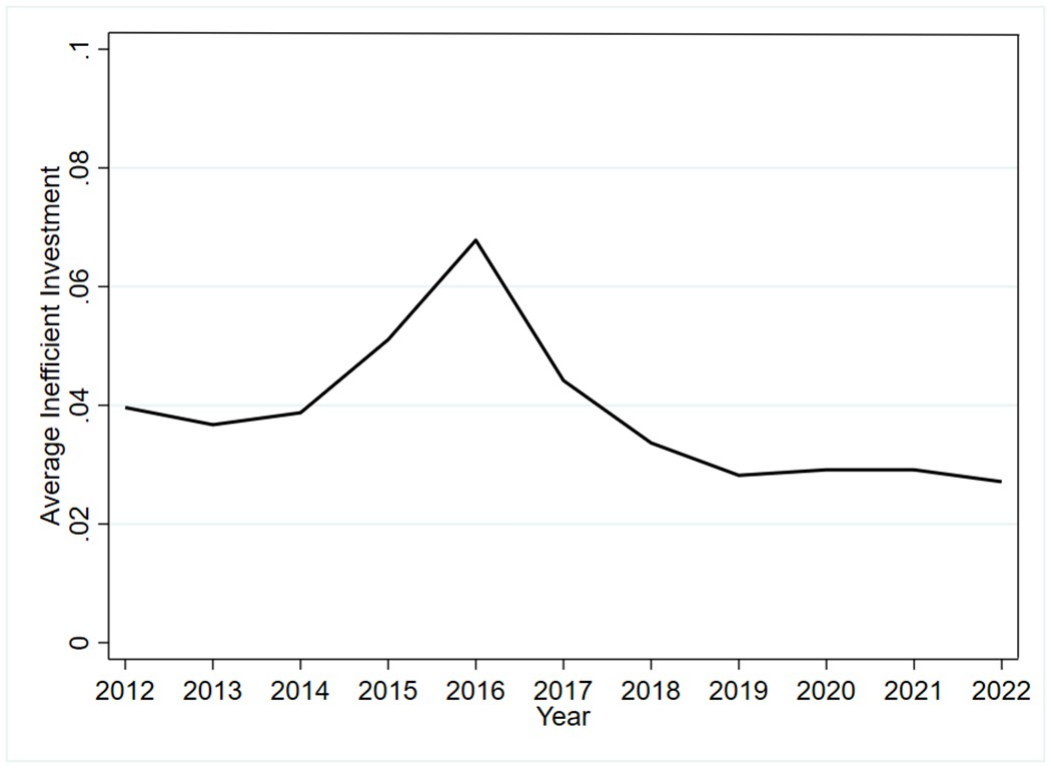
The trend of average inefficient investment from 2012 to 2022.

Table 3 shows the correlation analysis of the main variables, Absinv and TESTGFP have a negative and significant correlation, indicating an improvement in investment efficiency, which initially supports H1. The control variables and the explanatory variables are generally significant at the 1% level, demonstrating a correlation. The correlation coefficients between the explanatory variables, control variables, and interpreted variables are all less than 0.5, indicating that there is no serious multicollinearity among them. Table 4 displays descriptive statistics of the main variables. The minimum values for inefficiency investment, over-investment, and under-investment are all 0.0003, with maximum values of 0.5500. The sample means are 0.0388, 0.0453, and 0.0344, respectively, showing discrepancies with the medians. This indicates significant differences among companies in terms of investment efficiency, over-investment, and under-investment, with a notable prevalence of over-investment issues among enterprises. The trend of investment efficiency from 2012 to 2022 is shown in Fig 1, the larger the value, the less efficient the corporate investment is. The worst corporate investment efficiency in 2016, and the gradual improvement of corporate investment efficiency after 2017.

**Table 3:** Correlation analysis.

**variant**	**1**	**2**	**3**	**4**	**5**	**6**	**7**	**8**	**9**	**10**
1.Absinv	1.000									
2.TEST×GFP	-0.040***	1.000								
3.Top	-0.051***	-0.079***	1.000							
4.Indep	0.019***	0.005	0.044***	1.000						
5.Lever	-0.089***	-0.011	0.101***	0.010	1.000					
6.BM	-0.106***	0.057***	0.033***	-0.036***	-0.415***	1.000				
7.Grow	0.018**	-0.033***	0.025***	0.024***	0.118***	-0.097***	1.000			
8.ATO	-0.081***	0.013*	0.058***	-0.025***	0.146***	-0.094***	-0.177***	1.000		
9.AGE	-0.141***	0.092***	0.003	-0.014*	0.312***	-0.028***	0.052***	0.062***	1.000	
10.EQ	0.114***	0.079***	-0.096***	0.016**	-0.104***	-0.037***	-0.010	0.016**	0.044***	1.000

**Table 4:** Descriptive stats.

Variant	Sample Size	Average Value	Standard Deviation	Minimum Value	Maximum Values
Absinv	19,649	0.0388	0.0510	0.0003	0.5500
Underinv	7,948	0.0453	0.0620	0.0003	0.5500
Overinv	11,701	0.0344	0.0413	0.0003	0.5500
Top	19,649	0.3412	0.1485	0.0711	0.7668
Indep	19,649	0.3754	0.0545	0.3000	0.6000
Lever	19,649	0.4343	0.1970	0.0467	0.8656
BM	19,649	0.3421	0.1584	0.0533	0.9030
Grow	19,649	0.3825	0.9980	-0.6854	10.2879
ATO	19,649	0.6239	0.4348	0.0688	3.1172
AGE	19,649	2.4922	0.5942	1.0986	3.4340
EQ	19,649	0.0242	0.0324	0.0007	0.3139

### 4.2 Baseline regression

To examine the impact of the green finance policy on the investment efficiency of enterprises in the pilot regions, a baseline regression was conducted based on Eq (1), and the results are presented in Table 5. Column (1) indicates the impact of the green finance policy on corporate investment efficiency without any variable control, and the TEST×GFP is significant. Column (2) indicates that after variable control, the TEST×GFP is -0.005 is also significant. According to Eq (2), investment levels were subdivided into overinvestment and underinvestment categories. If the policy implementation mitigates both overinvestment and underinvestment levels in the pilot regions, it further verifies that the policy enhances corporate investment efficiency in pilot areas. As shown in columns (3)-(4) of Table 5, TEST×GFP is negative and significant. The results indicate that the green finance policy improves investment efficiency in pilot zones, thereby confirming Hypothesis 1.

**Table 5:** Baseline regression.

Variant	(1)	(2)	(3)	(4)
	Absinv	Absinv	Overinv	Underinv
TEST×GFP	-0.0054***	-0.0059***	-0.0041**	-0.0082**
	(-2.88)	(-3.16)	(-2.28)	(-2.54)
Top		-0.0091***	-0.0050*	-0.0124**
		(-3.09)	(-1.89)	(-2.41)
Indep		0.0189**	0.0137**	0.0209
		(2.31)	(2.00)	(1.40)
Lever		-0.0097***	-0.0311***	0.0141**
		(-3.02)	(-11.06)	(2.40)
BM		-0.0317***	-0.0375***	-0.0224***
		(-9.85)	(-12.46)	(-3.88)
Grow		0.0015***	-0.0002	0.0039***
		(2.67)	(-0.31)	(3.64)
ATO		-0.0093***	-0.0037***	-0.0151***
		(-8.03)	(-3.59)	(-7.94)
AGE		-0.0074***	-0.0032***	-0.0147***
		(-7.34)	(-3.54)	(-8.30)
EQ		0.1752***	0.1720***	0.1836***
		(10.48)	(9.36)	(6.27)
Province fixed effect	Yes	Yes	Yes	Yes
Industry fixed effect	Yes	Yes	Yes	Yes
Year fixed effect	Yes	Yes	Yes	Yes
N	19,649	19,649	11,701	7,948
Adj R^2^	0.0752	0.1077	0.1547	0.1014

Note: t-values after firm-level clustering are in parentheses;

*** indicates p<1%,

** indicates p<5%, and

* indicates p<10%. Same table below.

### 4.3 Robustness check

#### 4.3.1 Parallel trend test

Parallel trend test to verify the existence of the same trend between the pilot region enterprises and non-pilot region enterprises, it indicates that prior to the implementation of the policy, the pilot region enterprises and non-pilot region enterprises do not have a significant difference in investment efficiency, the DID method is also proved to be an unbiased estimation.


$$Absinv_{i,t}=\alpha_{0}+\alpha_{1}\sum_{t=-5}^{5}TEST_{p}\times GFP_{t}+\rho X_{i,t}+\mu_{p}+\delta_{j}+\theta_{t}+\varepsilon_{i,p,j,t}$$ (3)

**Fig 2:**
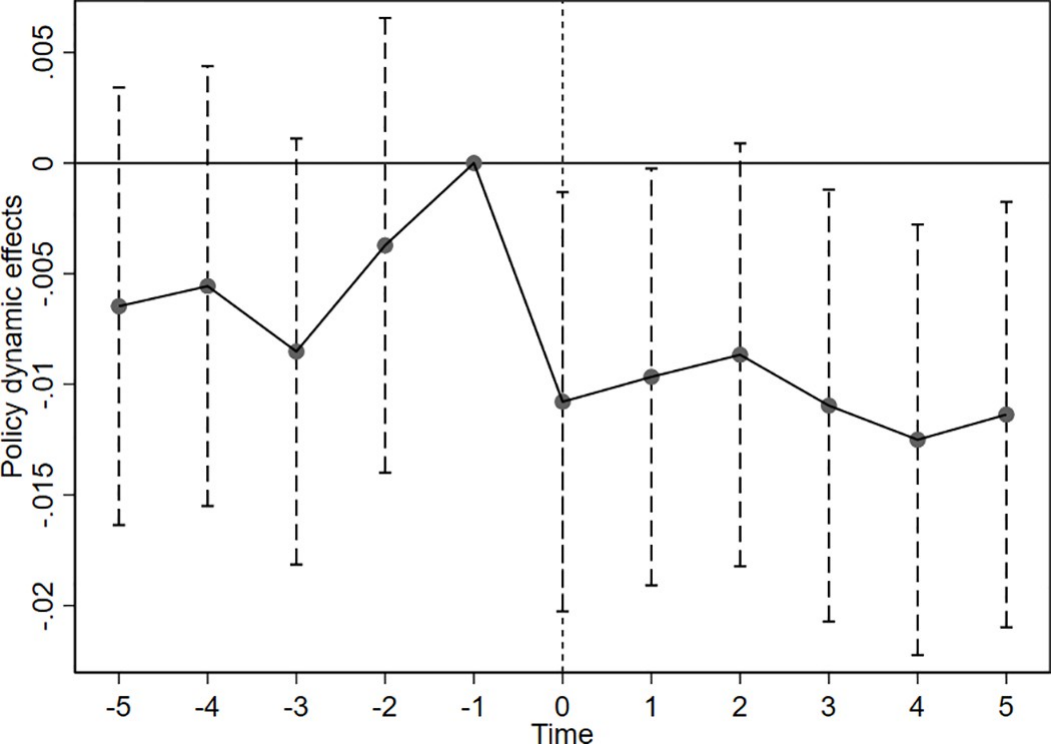
Parallel trend test.

To prevent multicollinearity, the year before the policy implementation (with 2016 as the base year) was excluded from the regression analysis. The results of the parallel trend test are shown in Fig 2 Before the implementation of the policy, the regression coefficient α_1_ and its 95% confidence interval does not significantly differ from 0, suggesting that there is no notable difference in the pre-policy trends between enterprises in the pilot region and those in the non-pilot region, which satisfies the parallel trend test. After the implementation of the policy, the regression coefficient α_1_ and its 95% confidence interval are gradually significantly different from 0, showing a downward trend, which demonstrates that the enhancement of investment efficiency in enterprises within the pilot region takes place after the implementation of the green finance policy. While there may be an initial lag in its effects, these benefits become increasingly evident over time.

#### 4.3.2 PSM-DID

Enterprises located in pilot zones are designated as the treatment group, and since the selection is not randomized, there may be individual differences between the pilot area enterprises and non-pilot area enterprises, which in turn leads to the endogeneity problem, so to reduce the impact of self-selection bias, PSM-DID is further used to enhance the unbiasedness of the results by robustness testing. Firstly, the control variables are used as covariates, and the probability of each sample becoming an enterprise in the pilot region is calculated by logit, secondly, according to the 1:1 near-neighbor matching method with put-back, the enterprises in the pilot region are matched to enterprises in the non-pilot region with similar propensity scores, and finally, the test is conducted. The results of the balance test can be found in Table 6, the deviation rate of the control variables of the enterprises in the pilot region and enterprises in the non-pilot region after propensity score matching is below 10%, with a p-value that is not statistically significant, so the matching results are ideal. The results of PSM-DID are displayed in Table 7, these findings align with the baseline regression results, with all regression coefficients for the interaction term TEST×GFP being less than 0 and significant at the 1% level, in the case of changing any of the fixed effects and the control variables. It is verified that after considering the influence of self-selection bias, the benchmark regression result is still robust.

**Table 6:** Balance test.

Variant	Unmatched	Mean		Bias	t-test
	Matched	TEST = 1	TEST = 0	%	p-value
Top	U	0.3298	0.3461	-11.1	0.000
	M	0.3299	0.3323	-1.7	0.353
Indep	U	0.3755	0.3754	0.1	0.964
	M	0.3755	0.3743	2.2	0.240
Lever	U	0.4205	0.4402	-10.1	0.000
	M	0.4205	0.4188	0.9	0.640
BM	U	0.3376	0.3440	-4.1	0.009
	M	0.3376	0.3405	-1.9	0.308
Grow	U	0.3490	0.3968	-4.9	0.002
	M	0.3490	0.3614	-1.3	0.463
ATO	U	0.6318	0.6206	2.6	0.098
	M	0.6318	0.6360	-1.0	0.605
AGE	U	2.4012	2.5308	-21.9	0.000
	M	2.4013	2.3914	1.7	0.377
EQ	U	0.0263	0.0233	8.8	0.000
	M	0.0262	0.0258	1.3	0.500

**Table 7:** PSM-DID test.

Variant	(1)	(2)	(3)	(4)
	Absinv	Absinv	Absinv	Absinv
TEST×GFP	-0.0097***	-0.0043*	-0.0062***	-0.0040*
	(-7.86)	(-1.75)	(-4.82)	(-1.69)
Controls	No	No	Yes	Yes
Province fixed effect	No	Yes	No	Yes
Industry fixed effect	No	Yes	No	Yes
Year fixed effect	No	Yes	No	Yes
N	8,851	8,851	8,851	8,851
Adj R^2^	0.0065	0.0765	0.0628	0.1119

#### 4.3.3 Placebo test

**Fig 3:**
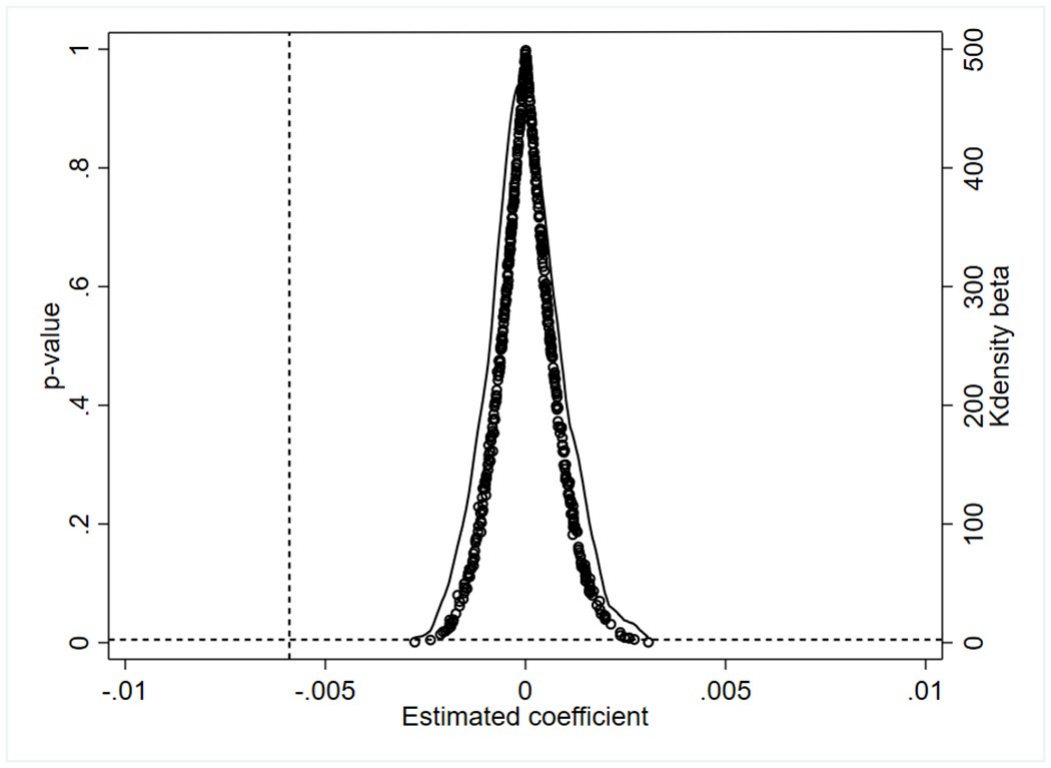
Placebo test.

To address concerns about the significant randomness of the baseline regression results and the potential endogeneity of the policy, a placebo test was conducted. A new treatment group was randomly selected with the same sample size as the original (5859), while the remaining samples were designated as the new control group, which was brought into Eq (1) to obtain the dummy TEST×GFP regression coefficients and p-values after 500 randomized simulated benchmark regressions. The test results are illustrated in Fig 3, where the regression coefficients from random simulations fall to the right of the baseline regression coefficients (-0.0059) and are concentrated around the value of 0. Most of the p-values obtained from random simulations are larger than the p-values obtained from the baseline regression (0.002) and are generally larger than the 0.1 level of significance. The results passed the placebo test, verifying that the finance policy does not have a significant effect on the investment efficiency of the dummy treatment group, and that the results are outliers, which do not occur randomly.

#### 4.3.4 Excluding other event interventions

First, the influence of other environmental regulations is excluded by control variables, and competing policies with similar enactment year and the green finance policy are selected; the promulgation of the “Environmental Protection Law of the People’s Republic of China” in 2015, which is called the most stringent in history, the enaction of the “Guiding Opinions on Building a Green Financial System” in 2016, which is known as the first year of green financial development, as well as the 2018 “Environmental Protection Tax Law” were enacted, all of which had an impact on the investment efficiency of enterprises. Considering that the above policies focus on heavy polluters, heavy polluters are selected to cross-multiply with the time of policy implementation to form an interaction term, which is substituted into the benchmark regression, and the results can be found in column (1) of Table 8. The outbreak of the 2020 Xin Guan epidemic shocks the investment decisions of enterprises to a certain degree, and to ensure that the results of the study are not interfered with by other external shocks. To ensure that the research results are not disturbed by other external shocks, the sample interval is narrowed and the benchmark regression is carried out after excluding the sample size in 2020, and the results can be found in column (2) of Table 8, the coefficient is still significantly negative. The time window period is shortened to two years before and after the green finance policy, i.e., 2015 to 2019, to reduce the impact of other policies on corporate investment efficiency. The regression results are presented in column (3) of Table 8.

**Table 8:** Other robustness tests.

Variant	(1)	(2)	(3)	(4)
	Absinv	Absinv	Absinv	Absinv
TEST×GFP	-0.0053***	-0.0058***	-.0086***	-0.0034**
	(-2.90)	(-3.12)	(-2.59)	(-2.38)
Controls	Yes	Yes	Yes	Yes
Province fixed effect	Yes	Yes	Yes	Yes
Industry fixed effect	Yes	Yes	Yes	Yes
Year fixed effect	Yes	Yes	Yes	Yes
N	19,649	17,832	9,317	19,649
Adj R^2^	0.1102	0.1080	0.1173	0.0389

#### 4.3.5 Replace the explanatory variables

Drawing on [61], investment efficiency is recalculated based on the growth rate of operating income and modeled as follows.


$$Invest_{i,t}=\beta_{0}+\beta_{1}Growth_{i,t-1}+\nu_{i,t}$$ (4)

Drawing on [62], the level of new investment (Invest) is defined as the ratio of capital expenditure to total assets. The measurement variable of Growth is changed from TobinQ to the growth rate of operating revenue, and the new residual is obtained after regression, and its absolute value is recorded as the level of inefficient investment (Absinv). Bringing this variable into the model, the results are shown in column (4) of Table 8, which are consistent with the results above and are robust.

## 5 Further analysis

### 5.1 Analysis of impact mechanisms

To evaluate whether the green finance policy can mitigate financial resource misallocation and lower agency costs to enhance the investment efficiency of enterprises in pilot areas, drawing on [63], the impact of financial resource misallocation and agency costs on corporate investment efficiency has been studied by a wide range of scholars, and there has been a relatively clear causal relationship, so focusing on the effect of the policy on financial resource misallocation and agency costs, and establishing the following mechanism effect model based on the original The following mechanism effect model is established based on the benchmark model.

$$M_{i,t}=\alpha_{0}+\alpha_{1}TEST_{p}\times GFP_{t}+\rho X_{i,t}+\mu_{p}+\delta_{j}+\theta_{t}+\varepsilon_{i,p,j,t}$$ (5)

where M_i,t_ represents an indicator of the impact mechanism, including the misallocation of firms’ financial resources (FM) and agency costs (Agen), and the remaining variables are consistent with the baseline model.

#### 5.1.1 Financial resource misallocation

Through theoretical analysis, financial institutions allocate resources more inclined to short-cycle and high-return projects, the favor of the investment object is often based on the enterprise scale, the total amount of assets and other capabilities, the investment in green enterprises is faced with a long payback period, the amount of return is unknown and other issues, in contrast to the heavy pollution enterprises as a pillar of the country, with a large volume and stable operating base, so the financial resources are biased in favor of heavy pollution Therefore, financial resources favor heavily polluting enterprises. The green finance policy guides financial institutions to differentiate resource allocation and facilitates the allocation of financial resources towards high-quality enterprises, ultimately mitigating issues of both under-investment and over-investment. To test the role of the mechanism of financial resource misallocation, referring to [64], we construct the corporate financial resource misallocation (FM) index. The FM is determined by the ratio of an enterprise’s cost of capital utilization to the average cost of capital utilization within its industry, where the enterprise’s cost of capital utilization = interest expense / (liabilities—accounts payable), and the result is taken as the absolute value. When the absolute value is larger, the greater the degree of financial resource misallocation, the smaller the corporate financial allocation efficiency, and vice versa. The result is shown in Table 9, The effect of the green finance policy on corporate investment efficiency was reassessed in the mechanism test due to the presence of sample size changes, column (2) illustrates the effect of the policy on financial resource misallocation, revealing a regression coefficient of -0.0555. This indicates that the green finance policy effectively mitigates the misallocation of financial resources among enterprises in the pilot region, which ultimately improves the efficiency of corporate investment. Hypothesis 2 is verified.

**Table 9:** Impact mechanism test results.

Variant	(1)	(2)	(3)	(4)
	Absinv	FM	Absinv	Agen
TEST×GFP	-0.0059***	-0.0555*	-0.0059***	-0.0052**
	(-3.17)	(-1.87)	(-3.16)	(-2.45)
Controls	Yes	Yes	Yes	Yes
Province fixed effect	Yes	Yes	Yes	Yes
Industry fixed effect	Yes	Yes	Yes	Yes
Year fixed effect	Yes	Yes	Yes	Yes
N	19,575	19,575	19,649	19,649
Adj R^2^	0.1074	0.1115	0.1077	0.4028

#### 5.1.2 Agency costs

Through theoretical analysis, the separation of powers and information asymmetry is the direct and essential cause of the agency conflict, shareholders are hindered from supervising the management of the enterprise, and Management makes unfavorable investment decisions due to the pursuit of private interests or consideration of private costs, the policy is customized to address the varying investment decisions to improve the corporate investment efficiency and break the asymmetry between the shareholders and the management of the enterprise through information sharing. Through information sharing, it breaks the asymmetry between shareholders and corporate management, improves information transparency and regulatory effectiveness, and promotes enterprises to make favorable decisions. To assess the mechanism of agency costs, the management expense ratio is employed as a proxy variable for agent costs. (Agen), which is calculated as the ratio of management expense to operating income, and the larger the value is, the higher the agency costs are, and vice versa, the lower it is. The regression results are presented in Table 9, column (4) is the effect of the green finance policy on agency costs. The regression coefficient is significant, indicating that agency costs serve as an important mechanism, and the policy can substantially lower these costs for enterprises in pilot areas, thereby enhancing investment efficiency in the pilot area. Hypothesis 3 is verified.

### 5.2 Heterogeneity analysis

To test for heterogeneity, a Difference-in-Differences-in-Differences (DDD) model is constructed as follows.


$$\begin{array}{c} Absinv_{i,t}=\alpha_{0}+\alpha_{1}TEST_{p}\times GFP_{t}\times Group_{i,t}+\alpha_{2}TEST_{p}\times GFP_{t}+\alpha_{3}Group_{i,t}+\rho X_{i,t}+\mu_{p}\\ \;+\delta_{j}+\theta_{t}+\varepsilon_{i,p,j,t} \end{array}$$ (6)

Group represents State-Owned Enterprises (SOE) and Research and Development (RD).

#### 5.2.1 State-owned enterprises (SOE)

First of all, state-owned enterprises, owing to their distinctive status and characteristics, serve a pillar role in China’s economic development, possess greater capital and more advanced technology, and are more capable of shouldering social responsibility and have a strong sense of environmental protection to ensure the sustainable development of state-owned enterprises. Before the implementation of the green finance policy, the state-owned enterprises play an exemplary role in making more effective investments in daily production and operations to strengthen the resilience for survival and development. Secondly, due to the great influence of state-owned enterprises, their production and operation are widely supervised by the government and society before the implementation of the policy, resulting in a lower degree of information asymmetry, so state-owned enterprises choose to improve the development mode and carry out more efficient investment in both active and passive. In addition, considering the problem of default risk, state-owned enterprises have more financing channels and alternative financing methods, and the degree of policy influence is lower. Under the differentiated allocation of financial resources and strengthened external supervision, non-state heavily polluting enterprises will gradually shift from the rough economic development mode to the green and low-carbon development mode to make more efficient investments. With the support and encouragement of the policy, the non-state-owned green enterprises in the pilot area will have sufficient funds to invest in the green industry, and the requirement of strengthening the transparency of environmental information reduces agency costs. Therefore, non-state-owned enterprises in the pilot area are more influenced by the policy. According to the nature of property rights to construct the cross-multiplier (TEST×GFP×SOE), the regression results are shown in column (1) of Table 10, the TEST×GFP is significant, This confirms that, under the policy, the investment efficiency of non-state-owned enterprises in the pilot areas will experience a more significant improvement.

**Table 10:** Heterogeneity analysis results.

Variant	(1)	(2)
	Absinv	Absinv
	Group = SOE	Group = RD
TEST×GFP×Group	0.0050**	0.0064***
	(2.40)	(3.07)
TEST×GFP	-0.0078***	-0.0096***
	(-3.91)	(-4.19)
Group	-0.0084***	-0.0043***
	(-6.99)	(-4.12)
Controls	Yes	Yes
Province fixed effect	Yes	Yes
Industry fixed effect	Yes	Yes
Year fixed effect	Yes	Yes
N	19,213	16,474
Adj R^2^	0.1119	0.1094

#### 5.2.2 Research and development (RD)

Research and Development is the embodiment of an enterprise’s core competence. Enterprises with better research and development ability can gain insights into market development trends and customer needs, and enhance their profitability and solvency with differentiated competitive strategies, while effectively reducing investment risks and increasing investment returns. Based on this, financial institutions are more inclined to extend financial support to enterprises that exhibit robust research and development capabilities, thereby promoting the efficient use of funds for value creation, so compared with enterprises with poor research and development ability, the investment efficiency of such enterprises is higher. Porter’s theory posits that appropriate environmental regulation can incentivize enterprises to invest in technological innovation to a certain degree, and innovation compensation can compensate for the increase in production costs. The green finance policy encourages enterprises to boost investments in research and development, and enterprises with poorer research and development ability can improve investment efficiency more significantly. To test the heterogeneous impact, research and development is selected as an indicator of technological innovation capacity, calculated as research and development expenditure expenditures plus 1 to take the logarithm. Firms in the sample that are higher than the industry average research and development are labeled as high-technology innovative enterprises and the rest are labeled as low research and development firms. The regression result can be found in column (2) of Table 10, the coefficient is positive, which confirms that under the implementation of the green finance policy, the investment efficiency of low research and development enterprises in the pilot region will be more significantly improved.

## 6. Discussion

Firstly, the study demonstrates that the green finance policy significantly enhances corporate investment efficiency, thereby supporting H1. This result is consistent with existing studies [54, 65]. Compared with previous studies [52] that focused only on heavily polluting enterprises, [32] that focused only on green enterprises, and [65] that validated that the green finance policy can promote corporate investment efficiency, but could not significantly alleviate under-investment, this paper addresses these gaps by expanding the scope of to include all enterprises. Moreover, this paper explores the effects of the green finance policy on both green enterprises and heavy polluters, further dividing investment efficiency into over-investment and under-investment. The findings indicate that the green finance policy can mitigate both over-investment and under-investment behaviors among enterprises, thereby enhancing investment efficiency in the pilot areas.

Secondly, the study identifies that the transmission mechanism of the green finance policy enhances corporate investment efficiency, this paper utilizes resource allocation theory and agency theory as frameworks, it highlights financial resource allocation and agency costs as the role of the mechanism, which supports H2 and H3. It has been confirmed in the literature that the green finance policy can optimize the allocation of resources, directing the flow of resources to high-efficiency enterprises, while also urging the financial institutions to leverage their governance effectiveness and the effectiveness of large lenders to regulate and screen enterprises. This includes building information-sharing platforms to alleviate information asymmetry [44, 66, 67]. Financial resources as an important element of enterprise investment, the efficiency of financial resource allocation affects the investment efficiency of enterprises, and the separation of powers and information asymmetry also affect the investment efficiency of enterprises [25, 68]. Based on the existing theoretical studies, this paper concludes that the green finance policy improves investment efficiency by alleviating financial resource misallocation and reducing agency costs.

Finally, the study reveals that the green finance policy has a more pronounced effect on non-state-owned enterprises and low-tech innovative enterprises. Consistent with the existing literature, state-owned enterprises and high-technology innovative enterprises generally have higher investment efficiency due to their financial advantages, credit advantages, and competitive advantages [69, 70], and unlike the literature on heterogeneity studies in the context of relevant policy [71, 72], this paper finds that non-state-owned enterprises and low-technology innovative enterprises are more policy-sensitive to the green finance policy, are more affected by the policy, and are more significant in terms of investment efficiency improvement.

## 7 Conclusions

### 7.1 Theoretical and practical significance

Firstly, theoretical significance. Existing literature on the microeconomic benefits of green finance mostly explores the content of corporate financing and corporate innovation [19, 48], with less research on corporate investment efficiency and divergence in viewpoints [32, 33, 35], this paper adds to the literature by emphasizing the beneficial effects of green finance policy on the effective utilization of corporate resources and orderly expansion of capital. It further confirms that under the synergistic effect of government regulation and market adjustment, the green finance policy greatly enhances corporate investment efficiency in pilot areas, alleviates the situation of over-investment and under-investment by enterprises, by reconciling economic development and environmental governance, promotes enterprises to maximize value creation. Moreover, existing literature lacks depth in exploring mechanisms of green finance’s effect on corporate investment efficiency, this paper is based on resource allocation theory and agency theory, an in-depth analysis of financial resources mismatch and agency costs of the mechanism of the role, while further refinement of the research object. It provides reference and theoretical support for enterprises to realize green and low-carbon development.

Secondly, practical significance. At the enterprise level, the phenomena of over-investment and under-investment are widespread among enterprises in China [8], Investigating how the green finance policy affects corporate investment efficiency provides valuable insights for enterprises to address issues of inefficient resource utilization and disorderly expansion. By vigorously developing green finance, and ensuring rational allocation of financial resources to efficient enterprises, enterprises can be prompted to prioritize environmental protection in their daily operations, establish a green reputation, and reduce managerial opportunism. This, in turn, leads to efficient investments, thereby providing a reference for increasing financial support for Entity economic development. At the policy level, the paper evaluates the implementation impact of the location-oriented green finance policy through the lens of investment efficiency, offering insights for the broader promotion of green policy across the country. The analysis of heterogeneous enterprises, aids local governments in refining the relevant standards for green finance and adjusting policy strategies promptly during the pilot process, offering a reference for the green finance policy to be tailored to local conditions and unleash their effectiveness.

### 7.2 Recommendations

Firstly, enhance the guiding role of the government. (1) Enhance the top-level design of green finance along with its supporting measures. The government should strategically coordinate and plan the development of green finance, expedite the development of the green financial system, improve the green financial risk prevention and compensation mechanism, and other supporting measures to safeguard corporate investment against risks. (2) Leverage the incentive and constraint mechanisms of the green finance policy. This involves directing financial resources and social capital to high-quality enterprises and green industries, encouraging enterprises to realize high-efficiency investment. Additionally, providing a conducive environment for optimizing the investment structure of enterprises by improving the financial regulatory system and strengthening the government’s supervision, rewards, and penalties for financial institutions in carrying out their business and services. (3) Expand the pilot zone appropriately and develop green finance according to local conditions. By refining and learning from pilot zone experiences, the government can enhance the universality of green finance policy by broadening the scope. Considering regional differences, local governments should adopt green finance strategies that are tailored to the local environment, guided by a framework of green finance standards. At the same time, differentiated green finance assessment indicators should be formulated to target and coordinate the development of heterogeneous enterprises.

Secondly, strengthen the role of the market mechanism. (1) Avoid one-size-fits-all restrictions and increase financial institutions’ support for enterprises in transition. Simply imposing green transformation requirements on “two high” enterprises without considering sustainable development may deviate from the purpose of green finance implementation. Financial institutions should, according to the progress of transformation and risk level of enterprises in transition, dynamically assess enterprise risks and timely adjust investment strategies, guide social capital to support enterprises in transition, and avoid the lack of operating funds for enterprises in transition, resulting in investment difficulties and eventual deterioration of operations. (2) Establish a diversified green financial market. Financial institutions actively advance the development of green finance through the innovation of green financial products and the creation of differentiated loan schemes, providing diversified financing channels to stimulate enterprises to optimize investment. Utilizing information-sharing platforms and credit rating indicators will help monitor enterprise investments in green projects and promote efficient allocation of resources.

Thirdly, leverage the subjective initiative of enterprises. (1) Establish an incentive and constraint mechanism linked to management performance. This mechanism should encourage management to pay attention to long-term interests and actively embrace social responsibility, thereby alleviating the corporate inefficiencies in investment caused by agency conflict. (2) Enhance the disclosure of environmental information. Enterprises disclose environmental information to reduce information asymmetry inside and outside the enterprise, address the problems of adverse selection and moral hazard, establish a green reputation, and transmit benign development signals to stakeholders, thus obtaining more financing support to protect corporate investment.

### 7.3 Limitations and future research directions

Firstly, there are limitations regarding the selection of the research sample. Given that the sample period extends to 2022 and considering the relatively short establishment time of the second batch of pilot zones, and it is difficult for the policy effect to appear, so this paper only studies the first batch of pilot zones. As the implementation time of the policy increases, future research will consider adding the second batch of pilot zones to expand the treatment group capacity.

Secondly, the limitations of research method selection. A major reason for choosing the DID method is that it is not possible to accurately know the actual data on the green finance policy support received by each sample company, so an exogenous policy is chosen as an explanatory variable, and the accuracy of data matching may be affected. With the continuous improvement of green finance data disclosure, when the impact of the green finance policy on micro-enterprises can be captured more easily, the study can select more accurate models for research.

Thirdly, the limitations of removing exogenous event shocks. All policies enacted by China from 2012 to 2022 that have an impact on corporate investment efficiency cannot be excluded or accounted for as dummy variables, and the intensity of these policies is difficult to judge, so it is difficult to obtain a cleaner treatment effect. In future research, rather than confining ourselves to a single policy instrument, we should fully consider the overlapping effects of policies.

## Supporting information

S1 File (ZIP)
